# Reduction in Gastrointestinal Cancers in Cirrhotic Patients Receiving Rifaximin vs Lactulose Only Therapy for Hepatic Encephalopathy

**DOI:** 10.7759/cureus.35259

**Published:** 2023-02-21

**Authors:** Ankoor H Patel, You Li, Carlos D Minacapelli, Kaitlyn Catalano, Vinod Rustgi

**Affiliations:** 1 Department of Internal Medicine, Rutgers Robert Wood Johnson Medical School, New Brunswick, USA; 2 Department of Medicine, Division of Gastroenterology and Hepatology, Rutgers Robert Wood Johnson Medical School, New Brunswick, USA

**Keywords:** gastrointestinal cancers, cancer, cirrhosis, lactulose, rifaximin

## Abstract

Background

Rifaximin and/or lactulose therapy is widely used in cirrhotic patients for the prevention and treatment of hepatic encephalopathy. The incidence of gastrointestinal cancers in these patients on lactulose, rifaximin, and/or combination therapy is unknown. We investigated the possible effect of lactulose and rifaximin on cancer risk in patients with cirrhosis using the MarketScan database.

Methods

A retrospective cohort study was conducted using the Truven Health MarketScan Commercial Claims databases from 2007-2017. An index date was defined for each participant as the earliest date of cirrhosis diagnosis. A baseline period for each participant was defined as the 12 months prior to the first medication date while the study follow-up period represented the period from the initiation of the medication to its cessation. ANOVA was used to compare all continuous measures of age and duration of medication. Wald Chi-square tests were performed to test the associations between the study groups.

Results

A total of 12,409 patients were included in our study. The rifaximin only cohort had the greatest reduction in risk of developing colon cancer, esophageal cancer, and stomach cancer compared to the other groups. Rifaximin reduced the risk of colon cancer and esophageal cancer by 59.42% and 70.37%, respectively, compared to patients taking lactulose only. Patients in the lactulose plus rifaximin cohort had the highest rate of development of pancreatic cancer (lactulose plus rifaximin vs rifaximin only vs lactulose only, 0.45% vs 0.24% vs 0.21%; P < 0.0001) and liver and intrahepatic bile duct cancers (11.73% vs 5.84% vs 5.49%; P < 0.0001).

Conclusion

Colon, esophageal, and gastric cancers had a marked incidence reduction in the rifaximin only cohort compared to the other cohorts studied.

## Introduction

Cirrhosis is a significant cause of mortality and morbidity worldwide with the liver disease ranking 11th and 15th as the leading cause of death and cause of morbidity, respectively. In 2016, cirrhosis accounted for 2.2% of deaths and 1.5% of disability-adjusted life years worldwide [1]. The increasing prevalence of cirrhosis worldwide, including USA, over the last decades is predominantly due to increasing rates of nonalcoholic fatty liver disease (NAFLD)/nonalcoholic steatohepatitis (NASH), and alcoholic liver disease while there have been decreasing rates of viral hepatitis [2-4]. Cirrhosis is associated with higher rates of hospitalization, readmissions, longer length of hospitalization, and worse outcomes in comparison with other disease states [5,6]. Healthcare utilization and costs associated with cirrhosis have been rising over the last two decades [7-9].

Complications of cirrhosis include hepatic encephalopathy (HE). Cirrhosis is also a primary risk factor for hepatocellular carcinoma (HCC), which has been increasing in incidence over the past three decades [10]. The prevalence of upper gastrointestinal (GI) cancer in patients with cirrhosis is higher compared to patients without cirrhosis [11,12]. Zullo et al. [12] showed a significant 2.6-fold (p < 0.01) increased prevalence of gastric cancer in patients with cirrhosis. Patients with cirrhosis have also been shown to have an increased risk of esophageal cancer and pancreatic cancer [13]. Results from a systematic review and meta-analysis by Komaki et al. showed an increased risk of colorectal carcinoma (CRC) in patients with chronic liver diseases and cirrhosis compared to the general population, a finding that persisted following liver transplantation [14].

Lactulose is a non-absorbable synthetic disaccharide that is commonly used in patients with cirrhosis to prevent and treat hepatic encephalopathy. Rifaximin is a synthetic, non-absorbable antimicrobial agent, used to treat and prevent HE via its broad-spectrum bactericidal activity against ammonia-producing colonic bacteria. Several studies and meta-analyses have shown the efficacy of lactulose in the prevention and treatment of HE [15-17]. Rifaximin has also been shown to have a beneficial effect on HE. A meta-analysis, which included 19 randomized control trials and 1370 patients, showed that rifaximin was efficacious in secondary prevention of HE, recovery from HE, and led to a reduction in mortality [18]. The use of combination lactulose and rifaximin therapy has been shown to be clinically superior compared to lactulose only therapy in recent studies. A few meta-analyses have shown that combination therapy increased clinical efficacy and decreased mortality, risk of hospitalization, length of stay, and complications of portal hypertension [19-22].

The aim of our study was to assess the development of various GI cancers in patients with cirrhosis who were administered lactulose, rifaximin, or combination therapy using the Truven® Commercial Claims (MSCC) database. It was hypothesized that effects on the gut microbiome might lead to different incidence rates of GI cancers in patients receiving therapy for HE.

## Materials and methods

Data source

We conducted a cohort study using the Truven Health MarketScan® Commercial Claims (MSCC) database from January 1st, 2007, to December 31st, 2017. Data included in the MSCC database represent national healthcare records from the United States (US) government and public organizations, large employers, and health plans from more than 350 payors annually. The MSCC databases include longitudinal individual-level data for health insurance claims across inpatient, outpatient, and outpatient prescription drug services. All MSCC records are de-identified data that are compliant with all US patient confidentiality requirements. The Internal Review Board (IRB) of Rutgers Robert Wood Johnson Medical School (New Brunswick, USA) approved the protocol (IRB Title: Liver Disease - MarketScan; Protocol #Pro2020000877) of this study.

Study sample

Analyzed subjects included those with cirrhosis as defined by the International Classification of Disease, Ninth Revision (ICD-9) code 571.5, as well as the Tenth Revision (ICD-10) code K74.6, K74.60, K74.69 in either inpatient admissions or outpatient services. It then included the subset on either lactulose, rifaximin or combination treatment.

An index date was defined for each participant as the earliest date of a cirrhosis diagnosis. The first medication date was defined as either the first date of lactulose or the first date of rifaximin. Patients with the first medication date before index date were excluded from the study. Patients with a history of any cancer before the first medication date were excluded from the study. A baseline period for each participant was defined as the 12 months prior to the first medication date while the study follow-up period represented the period from the first medication date to the last medication date captured. All participants with at least 12 months of enrollment before the first medication date were included in the study.

Baseline demographics, including age, gender, the region of residence and the type of health insurance plan were obtained from the index date records. A comorbidity profile was measured for each participant during the baseline period using ICD-9 codes. Disease profile included participants’ status with ascites, spontaneous bacterial peritonitis and sepsis, septic shock, hepatic encephalopathy and variceal bleeding. In addition, a total weighted Charlson Comorbidity Index (CCI) score was calculated for each participant during the baseline period [23]. GI cancers included were colon, rectum and anus, esophagus, stomach, pancreas, liver and intrahepatic bile duct, and other GI organs (e.g., retroperitoneum, small intestine, extrahepatic biliary tract, ampulla of Vater, gallbladder).

Study variables

Statistical Analysis

We compared lactulose only versus rifaximin only treatment in the cirrhosis population. Next, we compared these groups to the combination of lactulose plus rifaximin treatment. Baseline characteristics and comorbidity profiles for those with lactulose only, rifaximin only and combination lactulose plus rifaximin treatment were calculated in means and proportions. Analysis of Variance (ANOVA) was used to compare all continuous measures of age and duration of medication. Wald Chi-square tests were performed to test the associations between the study groups.

## Results

Sample characteristics and comorbidity profiles

**Figure 1:**
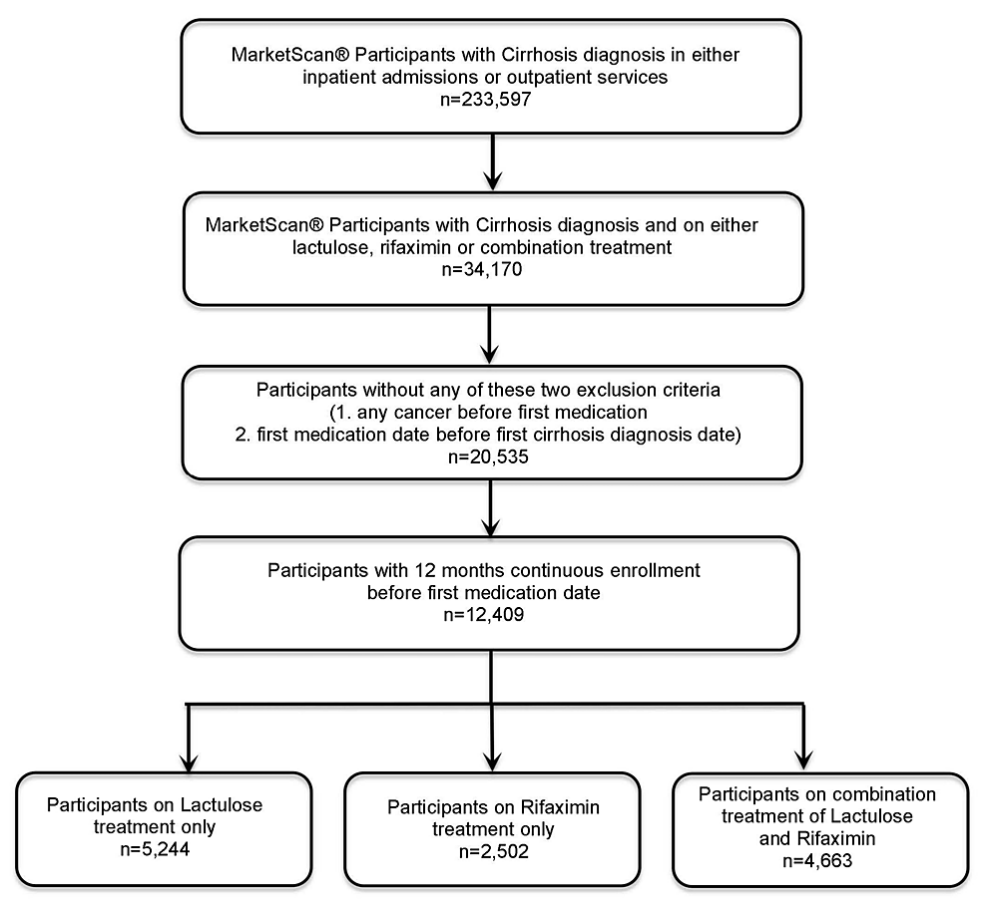
Diagram for study sample selection

The study sample included 34,170 participants from the MSCC database with cirrhosis and inclusion criteria of: age ≥ 18 years old, diagnosis of cirrhosis, and utilization of rifaximin, lactulose, or both medications. Of the 34,170 patients, 13,635 patients were excluded because either there was a diagnosis of cancer prior to the first use of medication or the date of the first use of the medication was before the first diagnosis date of diagnosis of cirrhosis. Of the study population of 12,409 patients, 5,244 patients were taking lactulose only, 2,502 were taking rifaximin only, 4,663 were taking both lactulose and rifaximin. Of this last group, 3,380 were taking both medications; however, the start of rifaximin followed the start of using lactulose. Patients were only included in the statistical analysis if the utilization of lactulose and rifaximin occurred after the date of their cirrhosis diagnosis (Figure 1).

Between-group differences of baseline characteristics are summarized in Table 1. Patients in the lactulose plus rifaximin group were generally older and had a higher proportion of male gender (% male, lactulose only vs rifaximin only vs lactulose plus rifaximin, 59.32% vs 57,75% vs 62.00%; P < 0.0001) compared to the lactulose only and rifaximin only cohorts. Patients in the rifaximin only cohort had the highest degree of comorbidities followed by lactulose only and last, lactulose plus rifaximin cohorts (CCI 4+, lactulose only vs rifaximin only vs lactulose plus rifaximin, 7.11% vs 7.67% vs 5.66%; P < 0.0001). A history of smoking was more common in the lactulose only cohort compared to the other two groups (% smoking, lactulose only vs lactulose plus rifaximin vs rifaximin only, 23.97% vs 20.87% vs 20.78%; P < 0.0001). Alcohol use disorder was most common in the lactulose only cohort followed by the lactulose plus rifaximin cohort (% alcohol use disorder, lactulose only vs lactulose plus rifaximin vs rifaximin only, 36.40% vs 34.85% vs 29.50; P < 0.0001). Patients in the lactulose plus rifaximin cohort had a longer mean duration of medication use compared to the rifaximin only and lactulose only cohorts (Mean duration of medication [days], 550.02 vs 210.87 vs 180.63; P < 0.0001) (see Table 1).

**Table 1:** Baseline characteristics of the study sample of patients by group

	Total	Lactulose only	Rifaximin only	Lactulose only vs Rifaximin only	Lactulose plus Rifaximin combination therapy	Lactulose only vs Rifaximin only vs Lactulose plus Rifaximin	Lactulose only vs Rifaximin only vs Lactulose plus Rifaximin
Patient Characteristics	n=12,409	n=5,244	n=2,502	p-value	n=4,663	p-value	(ANOVA) p-value
Age [Mean (SD)]	54.24 (8.12)	54.25 (8.15)	53.98 (9.18)	0.2063	54.37 (7.45)	<0.0001	0.1558
Age Group [n (%)]				0.0001		<0.0001	
<18	51 (0.41)	19 (0.36)	22 (0.88)		10 (0.21)		
18-34	292 (2.35)	128 (2.44)	80 (3.20)		84 (1.80)		
35-44	967 (7.79)	406 (7.74)	208 (8.31)		353 (7.57)		
45-54	3,925 (31.63)	1,694 (32.30)	709 (28.34)		1,522 (32.64)		
55+	7,174 (57.81)	2,997 (57.15)	1,483 (59.27)		2,694 (57.77)		
Gender [n (%)]				0.1889		0.0009	
Male	7,447 (60.01)	3,111 (59.32)	1,445 (57.75)		2,891 (62.00)		
Female	4,962 (39.99)	2,133 (40.68)	1,057 (42.25)		1,772 (38.00)		
Region of Residence [n (%)]				<0.0001		<0.0001	
Northeast	1,865 (15.03)	698 (13.31)	457 (18.27)		710 (15.23)		
North Central	2,542 (20.49)	1,083 (20.65)	557 (22.26)		902 (19.34)		
South	5,612 (45.23)	2,233 (42.58)	1,182 (47.24)		2,197 (47.12)		
West	2,156 (17.37)	1,110 (21.17)	273 (10.91)		773 (16.58)		
Unknown	234 (1.89)	120 (2.29)	33 (1.32)		81 (1.74)		
Type of Health Insurance [n (%)]				<0.0001		<0.0001	
Preferred Provider Organization	7,417 (59.77)	3,093 (58.98)	1,540 (61.55)		2,784 (59.70)		
Health maintenance organization	1,612 (12.99)	764 (14.57)	249 (9.95)		599 (12.85)		
Comprehensive	535 (4.31)	260 (4.96)	110 (4.40)		165 (3.54)		
Point-of- Service with Capitation	1,023 (8.24)	397 (7.57)	200 (7.99)		426 (9.14)		
Other	1,822 (14.68)	730 (13.92)	403 (16.11)		689 (14.78)		
BMI				0.8728		0.9157	
18 - ≤25	83 (11.64)	29 (11.24)	26 (13.47)		28 (10.69)		
25-30	138 (19.35)	48 (18.60)	41 (21.24)		49 (18.70)		
30-35	130 (18.23)	51 (19.77)	35 (18.13)		44 (16.79)		
35-40	91 (12.76)	34 (13.18)	24 (12.44)		33 (12.60)		
40+	271 (38.01)	96 (37.21)	67 (34.72)		108 (41.22)		
Smoking [n (%)]	2,750 (22.16)	1,257 (23.97)	520 (20.78)	0.0018	973 (20.87)	0.0002	
Alcohol use disorder [n (%)]	4,272 (34.43)	1,909 (36.40)	738 (29.50)	<0.0001	1,625 (34.85)	<0.0001	
Duration of medication, Mean (SD)	325.54 (471.42)	180.63 (347.14)	210.87 (343.66)	0.0003	550.02 (558.15)	<0.0001	<0.0001
Charlson Comorbidity Index [n (%)]				<0.0001		<0.0001	
0	4,456 (35.91)	2,002 (38.18)	825 (32.97)		1,629 (34.93)		
1	3,871 (31.20)	1,599 (30.49)	775 (30.98)		1,497 (32.10)		
2	2,185 (17.61)	870 (16.59)	449 (17.95)		866 (18.57)		
3	1,068 (8.61)	400 (7.63)	261 (10.43)		407 (8.73)		
4+	829 (6.68)	373 (7.11)	192 (7.67)		264 (5.66)		
Ascites [n (%)]	6,918 (55.75)	2,675 (51.01)	1,370 (54.76)	0.0020	2,873 (61.61)	<0.0001	
Spontaneous bacterial peritonitis [n (%)]	599 (4.83)	203 (3.87)	138 (5.52)	0.0010	258 (5.53)	<0.0001	
Sepsis [n (%)]	894 (7.20)	355 (6.77)	221 (8.83)	0.0012	318 (6.82)	<0.0001	
Septic Shock [n (%)]	255 (2.05)	9 (1.83)	66 (2.64)	0.0203	93 (1.99)	<0.0001	
Hepatic encephalopathy [n (%)]	3,778 (30.45)	1,235 (23.55)	772 (30.86)	<0.0001	1,771 (37.98)	<0.0001	
Variceal Bleeding [n (%)]	2,014 (16.23)	730 (13.92)	395 (15.79)	0.0292	889 (19.06)	<0.0001	

History or development of disease

The development of complications due to decompensated cirrhosis among the cohorts is represented in Table 1 and Table 2. Historically, a higher proportion of patients in the lactulose plus rifaximin cohort developed complications of decompensated cirrhosis compared to the other cohorts while sepsis and septic shock was more commonly diagnosed in the rifaximin cohort (Table 1). The incidence of complications and comorbidities in the follow-up period following the index date is listed in Table 2. Patients in the lactulose plus rifaximin cohort had a higher comorbidity burden (as evidenced by the CCI) and had a higher rate of developing complications of decompensated cirrhosis (Table 2).

**Table 2:** Progression of disease and complications of the cohorts in the follow-up period

	Total	Lactulose only	Rifaximin only	Lactulose only vs Rifaximin only	Lactulose plus Rifaximin combination therapy	Lactulose only vs Rifaximin only vs Lactulose plus Rifaximin
Patient Characteristics	n=12,409	n=5,244	n=2,502	p-value	n=4,663	p-value
Charlson Comorbidity Index [n (%)]				<0.0001		<0.0001
0	3,013 (24.28)	1,685 (32.13)	640 (25.58)		688 (14.75)	
1	3,489 (28.12)	1,553 (29.61)	731 (29.22)		1,205 (25.84)	
2	2,527 (20.36)	978 (18.65)	492 (19.66)		1,057 (22.67)	
3	1,629 (13.13)	509 (9.71)	342 (13.67)		778 (16.68)	
4+	1,751 (14.11)	519 (9.90)	297 (11.87)		935 (20.05)	
Ascites [n (%)]	8,094 (65.23)	2,909 (55.47)	1,534 (61.31)	<0.0001	3,651 (78.30)	<0.0001
Spontaneous bacterial peritonitis [n (%)]	1,050 (8.46)	266 (5.07)	183 (7.31)	<0.0001	601 (12.89)	<0.0001
Sepsis [n (%)]	1,380 (11.12)	427 (8.14)	277 (11.07)	<0.0001	676 (14.50)	<0.0001
Septic Shock [n (%)]	391 (3.15)	112 (2.14)	88 (3.52)	0.0003	191 (4.10)	<0.0001
Hepatic encephalopathy [n (%)]	5,745 (46.30)	1,632 (31.12)	953 (38.09)	<0.0001	3,160 (67.77)	<0.0001
Variceal Bleeding [n (%)]	2,623 (21.14)	832 (15.87)	470 (18.78)	0.0013	1,321 (28.33)	<0.0001

Incidence or development of GI cancers

The incidence of GI cancers in the analyzed cohorts is listed in Table 3. The GI cancers studied were colon, rectum and anus, esophageal, stomach, pancreas, liver and intrahepatic bile duct, and other GI organs (e.g., retroperitoneum, small intestine, extrahepatic biliary tract, ampulla of Vater, gallbladder). Patients in the rifaximin only cohort had a decreased risk of colon cancer compared to the lactulose plus rifaximin and lactulose only cohorts (rifaximin only [n = 2,502] vs lactulose plus rifaximin [n = 4,663] vs lactulose only [n = 5,244], 0.28% vs 0.56% vs 0.69%; P < 0.0001). Compared to the lactulose only and lactulose plus rifaximin cohorts, patients in the rifaximin only cohort had a lower rate of developing esophageal (0.08% vs 0.19% vs 0.27%; P < 0.0001) and stomach (0.00% vs 0.15% vs 0.31%; P < 0.0001) cancers. In comparison with the rifaximin only cohort and lactulose only cohort, patients in the lactulose plus rifaximin cohort had the highest rate of pancreas (Lactulose plus rifaximin vs rifaximin only vs lactulose only, 0.45% vs 0.24% vs 0.21%; P < 0.0001), liver and intrahepatic bile duct (11.73% vs 5.84% vs 5.49%; P < 0.0001), and other GI organ cancers (0.47% vs 0.28% vs 0.27%; P < 0.0001).

**Table 3:** Development of GI cancer in the Lactulose only, Rifaximin only, and Lactulose plus Rifaximin cohorts in the follow-up period.

	Total	Lactulose only	Rifaximin only	Lactulose only vs Rifaximin only	Lactulose plus Rifaximin combination therapy	Lactulose only vs Rifaximin only vs Lactulose plus Rifaximin
Patient Characteristics	n=12,409	n=5,244	n=2,502	p-value	n=4,663	p-value
Total GI Cancers [n (%)]	1,114 (8.98)	350 (6.67)	168 (6.71)	0.9470	596 (12.78)	<0.0001
Colon [n (%)]	69 (0.56)	36 (0.69)	7 (0.28)	0.0243	26 (0.56)	<0.0001
Rectum and anus [n (%)]	23 (0.19)	10 (0.19)	7 (0.28)	0.4334	6 (0.13)	<0.0001
Esophagus [n (%)]	25 (0.20)	14 (0.27)	2 (0.08)	0.0900	9 (0.19)	<0.0001
Stomach [n (%)]	23 (0.19)	16 (0.31)	0	0.0025	7 (0.15)	<0.0001
Pancreas	38 (0.31)	11 (0.21)	6 (0.24)	0.7916	21 (0.45)	<0.0001
Liver and intrahepatic bile duct [n (%)]	981 (7.91)	288 (5.49)	146 (5.84)	0.5389	547 (11.73)	<0.0001
Other GI organs; peritoneum [n (%)]	43 (0.35)	14 (0.27)	7 (0.28)	0.9193	22 (0.47)	<0.0001

## Discussion

To our knowledge, this is the first study to utilize real-life claims data to evaluate the development of GI cancers in patients with cirrhosis taking lactulose, rifaximin, or both medications concomitantly. This study showed the rifaximin cohort had the lowest rate of developing esophagus, stomach, and colon cancers but a higher likelihood of developing rectal and anal cancer. This was seen also in the combination of rifaximin/lactulose though there was a blunted effect statistically. Patients in the lactulose and rifaximin cohort had a statistically higher rate of developing pancreas, liver and intrahepatic bile duct, and other GI organ (e.g., retroperitoneum, small intestine, extrahepatic biliary tract, ampulla of Vater, gallbladder) cancers compared to the rifaximin and lactulose only cohorts.

The anti-cancer mechanisms of lactulose and rifaximin are poorly understood. Literature on the anti-cancer effects of lactulose is scarce and often, contradictory. Roncucci et al. showed a significant reduction in adenoma recurrence following lactulose supplementation, defined as 20g/daily for up to three years [24]. Lactulose may exert anti-cancer properties via interaction with galectins which typically bind to β-galactosides. Overexpression of many galectins, notably galectin-1 and galectin-3, occurs in cancerous cells and correlates with tumor progression and metastasis in some cancers [25-27]. On the other hand, lactulose may exert its anti-cancer properties via alteration of the gut microbiome. Using a mouse model, Fernandez et al. showed a significant reduction in the number of colon tumors in the cohort treated with galacto-oligosaccharide derivatives of lactulose. Interestingly, the treatment group showed restoration of microbial diversity and a shift towards beneficial anti-inflammatory bacteria [28].

In our study, the rifaximin cohort had the lowest rate of developing esophageal, gastric, and colon cancers. Certain antibiotics (e.g., doxycycline, fluoroquinolones, rifabutin, nitroxoline, etc.) have been shown to exhibit anti-cancer activity and have been studied in clinical trials [29-32]. Like many forms of cancer, including colorectal cancer (CRC), angiogenesis is an important pathway in tumorigenesis. Rifaximin has been shown to have anti-inflammatory and anti-neoplastic properties that may be attributed to the inhibition of nuclear kappa factor B (NF-κB) signaling and nitric oxide (NO) release via activation of intestinal human pregnane X (PXR) receptors. This has been demonstrated in humanized PXR mouse models, and in vitro in human intestinal and colorectal epithelium [33-35]. Using PXR-humanized mice, rifaximin has been shown to significantly decrease the number of colon tumors induced by azoxymethane (AOM) and dextran sulfate sodium (DSS) treatment, increase survival rate, and inhibit NF-κB compared to wild type or PXR-null mice [36]. Proliferating cell nuclear antigen (PCNA) is a biomarker of colorectal cancer and its expression is associated with malignancy, angiogenesis, and metastasis [37,38]. In an in vitro model using human colorectal epithelium, rifaximin also demonstrated a significant PXR-mediated inhibition in cell proliferation, migration, and PCNA expression as well as vascular endothelial growth factors (VEGF) secretion, NO release, and inhibition of NF-κB [35]. Further work is needed to understand a possible mechanism in explaining our findings.

Rifaximin may exert its anticancer properties via modulation of the gut microbiome. The use of rifaximin has been widely examined in cirrhosis and hepatic encephalopathy (HE), irritable bowel syndrome (IBS), small intestinal bacterial overgrowth (SIBO), traveler’s diarrhea, etc. Using murine models, rifaximin reduced pro-inflammatory cytokine production via alteration of microbiota composition [39,40]. Interestingly, in a murine model, rifaximin resulted in moderate reduction in HCC development in DEN-exposed animals via mechanisms involving intestinal microbiota and toll-like receptor 4 (TLR4) signaling [41]. However, further studies are needed to understand rifaximin’s anti-cancer properties and to investigate whether cancer development is impacted in patients on rifaximin for the reasons mentioned above.

Smoking and alcohol use disorder was significantly higher in the lactulose only cohort compared to the rifaximin only and lactulose plus rifaximin cohorts. Patients in the rifaximin cohort had the lowest rate of smoking (rifaximin only vs lactulose plus rifaximin vs lactulose only, 20.78% vs 20.87% vs 23.97%) and alcohol use disorder (29.50% vs 34.85% vs 36.40%) compared to the other cohorts. The lower proportion of smoking and alcohol use in the rifaximin cohort may have contributed to the reduction in certain GI cancers in this group.

Strengths/Limitations

This study has several strengths. The use of the MSCC database provides access to longitudinal data in the form of individual-level health insurance data from an administrative claims database. The use of a randomized index date in patients with continuous enrollment ensured capture of data and analysis of disease progression and development.

Limitations included the use of claims data which may not capture the entire clinical picture. Our follow-up period was defined as the period between the dates of first use and last use of the medication of interest. Therefore, the incidence of GI cancers may be underestimated if the disease of interest began after our defined study period. Another limitation of this study is the relatively short duration of medication use in our study. The mean duration of medication was 180.63, 210.87, and 550.02 days in the lactulose only, rifaximin only, and lactulose plus rifaximin cohorts, respectively. Furthermore, MSCC captures commercial claims which only represents the U.S. population with private health insurance. Thus, it may be difficult to generalize the results to un-insured or under-insured Americans.

## Conclusions

Our study shows the frequency and rate of developing certain GI cancers in patients with cirrhosis on therapy for hepatic encephalopathy with lactulose or rifaximin or a combination thereof. The incidence of colon, esophageal, and gastric cancer was lowest in the rifaximin only cohort and highest in the lactulose only cohort. Further data on the possible reduction in incidence of GI cancers in cirrhotic patients on HE therapy are warranted to gain a better understanding of any possible anti-cancer mechanism of lactulose and more importantly, rifaximin.
